# Problem-Based Learning – Experiencing and understanding the prominence during Medical School: Perspective

**DOI:** 10.1016/j.amsu.2019.09.004

**Published:** 2019-09-15

**Authors:** James Curtis Dring

**Affiliations:** a Wenzhou Medical University, Wenzhou, China; b Medical University of Lublin, Poland

**Keywords:** PBL, Problem-based learning, Students perspective, Medical education

## Abstract

The realm of medicine and medical schools will now know of the first dual medical program to obtain a Bachelor of Medicine and Surgery (MBBS) and Doctor of Medicine (MD). Clinical practise and problem-based learning (PBL) will unify students to commence working together and effectively communicate to construct a more patient-focused experience. PBL has changed a whole new perspective for learning and retaining the vital information. PBL progressed and put into clinical practise on real patients in the hospitals will undoubtedly enhance critical thinking and judgement in intense situations. Implementing PBL from the start of medical school will produce not only tacit clinical knowledge and judgement but provide the confidence and preparation needed to convey independence. Therefore to conclude all experiences of PBL and the results from a meta-analysis strongly suggest an imperative approach to the advancement into the medical curricula in the near future.

## Highlights

• Medical education utilising problem-based learning.
• Starting an opportunity for students in first year of medical school.
• Utilising resources and have practicality in teaching hospitals.
• Mentally preparing students to the world of medicine.
• Hands on training and exposure to patients in real life scenarios.

That who already completed or otherwise endured the study of medicine or dentistry knows the full force of what's expected. Not only studying the foundational, yet fundamental basics, which every student has to go through at any university studying any subject. This course that I am undertaking is the first of its kind, as a first year doing the worlds first dual degree (MBBS/MD – *Huatuo* ***华佗***) program at Wenzhou Medical University (China) and the Medical University of Lublin (Poland). Studying abroad is already pushing the boundaries of one's own limitations. Studying medicine in China is both exciting and intriguing; the world already knows what China has accomplished in such a short time, hence fourth studying in China is something to be an escapade. This will be the pinnacle of all medical exposures and teachings across the globe.

Early exposure at the First Affiliated Hospital,^1^ reminds not only myself but for every medical student on their journey. Exposing yourself to sick and critically ill patients had a toll on the psychology or otherwise the thoughts of empathic intentions of wanting to help, but do not know how or where to start. First-year students had the pleasure of attending some special clinical education and learning some basic clinical concepts of assessing real patients in this type of environment.

Other medical schools around the world do not expose their students to what exactly happens on the hospital wards. Instead, most students are thrown into the deep end, rather than being mentally trained and prepared. Some wait until their fourth or fifth year to gain access to the teachings from qualified and experienced registrars. Problem-based learning (PBL) may not be introduced until the clinical years as premature understandings of clinical features and pathologies occur in the basic medicine years of education. In this report, I provide a concise student's perspective on PBL and its appropriateness for medical education starting from the first year.

Problem-based learning was developed and created back in the 1960s at McMaster University with an innovative approach to modern medical education [1]. The current medical curriculum generated is combined with theory-based knowledge or Lecture-Based Learning (LBL) from the classroom, then applying what was learned to real-life situations and “acting” as the physician looking after said patient(s) either in clinical environments like the hospital or primary health care centres.

Problem-based learning is deemed to be the best approach to clinical medicine enabling us to dissect a particular problem and discussing with our peers (our supervisor was just listening rather than intervening) in a safe and appropriate environment. The cases denoted were some of the common diseases that many doctors face when presented in the hospital, for instance, a 52-year man with a long history of alcoholism presented to the emergency department with gastrointestinal bleeding. Compiling research and irrefutable resources from Pubmed, Google Scholar, and Cochrane Library these scenarios embark not only understanding and synergizing authentic situations but amalgamating basic scientific principles to clinical medicine, reviewing medical history and going through each section one by one with each student taking responsibility to gather up information.

At Wenzhou Medical University a small group of students (50% of my class who attended) was given the opportunity to experience PBL with a respected physician in the respiratory department from the First Affiliated Hospital. We begin by attending only a single class to understand how to construct PBL; indulging into our first case followed by isolating some learning criteria which neither of us heard before and why was it used on said patient. The dynamics of small symposium amongst our group was challenging and rather supportive knowing our strengths and weaknesses. PBL in China and their vast population incurred us to take our PBL sessions into the hospital and visit all the patients who been diagnosed or had recovered from such diseases. PBL sessions such as this are beyond any doubt utilising the resources to the fullest extent in medical education. For instance, I've felt heavily pregnant women whose been scheduled for a caesarean, it was a privilege to be able to check the position of the baby and examine the babies healthy heart via a sonogram.

The advantages of incorporating PBL into everyday learning is copiously gratifying and nurturing for students to the doctors of tomorrow. Consequently, PBL alone isn't as versatile due to the lack of anatomical and physiological mechanisms, certain subjects can be rather daunting like clinical biochemistry and pathology. Considering most first-year students have limited knowledge and experience since leaving high school as this may render personal growth in their development. Since being a graduate in Biomedical Science^2^ my fellow colleagues looked for me for guidance when running PBL sessions. My peers have commented on my presence of an “expert” which can often counteract the learning experience as I would take the lead and speak more often than none as some cases presented would not have all the relative information.

Based on the current literature and the findings from Ref. [2]; LBL has little influence on generic self-study, only if there is a distinct interest. The result of a Chinese study similarly validates the use of PBL incorporating into their curricula more than traditional lecture-based learning [3] as students truly benefit from solving a similar problem in their chosen field of expertise. Reviewing all the relevant literature I can achieve and concur that PBL has made a significant improvement in my overall learning experience, not only retaining the hands-on training from doctors in several wards but strive to improve my own professional development in the field of medicine.

To summarise all that I have encountered here in China and soon to be starting the MD program in Poland. PBL, the way that I was shown practising professional etiquette, reviewing the history prior and making a diagnosis with a treatment plan in place for the patient. This, I feel PBL directed in this way should be introduced either as a teachable class or at least an extra-curricular class from the start of medical school. PBL provides all students with the necessary tools that indefinitely enhance the skills for critical thinking, abstract analysis, and good decision making when diagnosing patients and reviewing judgement calls for what is right for the patient. As these requirements are critical to be a good doctor. All things considered, PBL has been a privilege for my classmates and I. Learning how to apply knowledge to complex problems is the basis for future advancements not only in science but as well as centring the focus on patient care as humanly as possible.

## Ethical approval

None required.

## Sources of funding

None.

## Author contribution

James C. Dring LIBMS conceived and wrote the manuscript (perspective piece).

## Consent

N/A.

## Registration of research studies

1. Name of the registry: N/A.

2. Unique Identifying number or registration ID: N/A.

3. Hyperlink to the registration (must be publicly accessible).

## Guarantor

James Curtis Dring, BSc Hons, LIBMS.

## Conflicts of interest

None.

## Footnotes

1 Is considered the largest hospital in the whole of Asia, in 2017, it handled nearly 4,414,000 outpatient & emergency visits, admit approximately 162,000 inpatients and performed more than 85,000 operations, ranking top in Zhejiang province [4].

2 I am a graduate from the School of Bioscience at the University of East London. I have a degree in Biomedical Science (Honors) and having a year in a clinical laboratory at one of the leading hospitals in England. I have experience in Blood Sciences, Clinical Chemistry, Point Of Care Testing, Haematology and Transfusion Science.
